# Hospital care for critical illness in low-resource settings: lessons learned during the COVID-19 pandemic

**DOI:** 10.1136/bmjgh-2023-013407

**Published:** 2023-11-02

**Authors:** Mike English, Jacquie Oliwa, Karima Khalid, Onesmus Onyango, Tamara Mulenga Willows, Rosanna Mazhar, Elibariki Mkumbo, Lorna Guinness, Carl Otto Schell, Tim Baker, Jacob McKnight

**Affiliations:** 1 KEMRI-Wellcome Trust Research Programme, Health Services Unit, Nairobi, Kenya; 2 Health Systems Collaborative, Nuffield Department of Medicine, University of Oxford, Oxford, UK; 3 Muhimbili University of Health and Allied Sciences, Dar es Salaam, United Republic of Tanzania; 4 Ifakara Health Institute, Ifakara, United Republic of Tanzania; 5 London School of Hygiene and Tropical Medicine, London, London, UK; 6 Centre for Global Development, London, UK; 7 Department of Global Public Health, Karolinska Institute, Stockholm, Sweden; 8 Centre for Clinical Research Sörmland, Uppsala University, Eskilstuna, Sweden; 9 Department of Medicine, Nyköping Hospital, Nyköping, Sweden; 10 Karolinska Institute, Stockholm, Sweden; 11 Department of Clinical Research, London School of Hygiene and Tropical Medicine, London, UK

**Keywords:** COVID-19, Health systems, Health services research, Hospital-based study

## Abstract

Care for the critically ill patients is often considered synonymous with a hospital having an intensive care unit. However, a focus on Essential Emergency and Critical Care (EECC) may obviate the need for much intensive care. Severe COVID-19 presented a specific critical care challenge while also being an exemplar of critical illness in general. Our multidisciplinary team conducted research in Kenya and Tanzania on hospitals’ ability to provide EECC as the COVID-19 pandemic unfolded. Important basic inputs were often lacking, especially sufficient numbers of skilled health workers. However, we learnt that higher scores on resource readiness scales were often misleading, as resources were often insufficient or not functional in all the clinical areas they are needed. By following patient journeys, through interviews and group discussions, we revealed gaps in timeliness, continuity and delivery of care. Generic challenges in transitions between departments were identified in the receipt of critically ill patients, the ability to sustain monitoring and treatment and preparation for any subsequent transition. While the global response to COVID-19 focused initially on providing technologies and training, first ventilators and later oxygen, organisational and procedural challenges seemed largely ignored. Yet, they may even be exacerbated by new technologies. Efforts to improve care for the critically ill patients, which is a complex process, must include a whole system and whole facility view spanning all areas of patients’ care and their transitions and not be focused on a single location providing ‘critical care’. We propose a five-part strategy to support the system changes needed.

**Summary box**

- At the onset of the COVID-19 pandemic critical care was too often narrowly conceptuallised as comprising skilled, technologically supported care delivered in specific hospital locations, as a result the response initially paid insufficient attention to Essential Emergency and Critical Care that is needed in all facilities and across all clinical locations in larger facilities
- Ensuring Essential Emergency and Critical Care is available in all clinical settings requires a change in mindset of managers, who must prioritise essential forms of care, and requires that all healthcare providers and teams develop a form of systemic vigilance and response that becomes central to their work.
- Achieving such system change will, we propose, require a systems response that we articulate as five key strategies to generate alignment of different actors' efforts combined with sustained, skilled leadership and management

## Introduction

In the early months of the COVID-19 pandemic, as surges in caseloads of critically ill patients caused by SARS-CoV2 infection threatened to overwhelm high-income country hospitals, a multidisciplinary, collaborative team of researchers was assembled. The team hoped to examine the provision of care for critical illness in Kenya and Tanzania in the face of major concerns about the consequences of fundamental gaps in capacity and care quality in less resourced countries’ hospitals. These had previously been highlighted in areas such as maternal, neonatal and child health, general adult and critical and perioperative care.^1–6^

**Figure 1:**
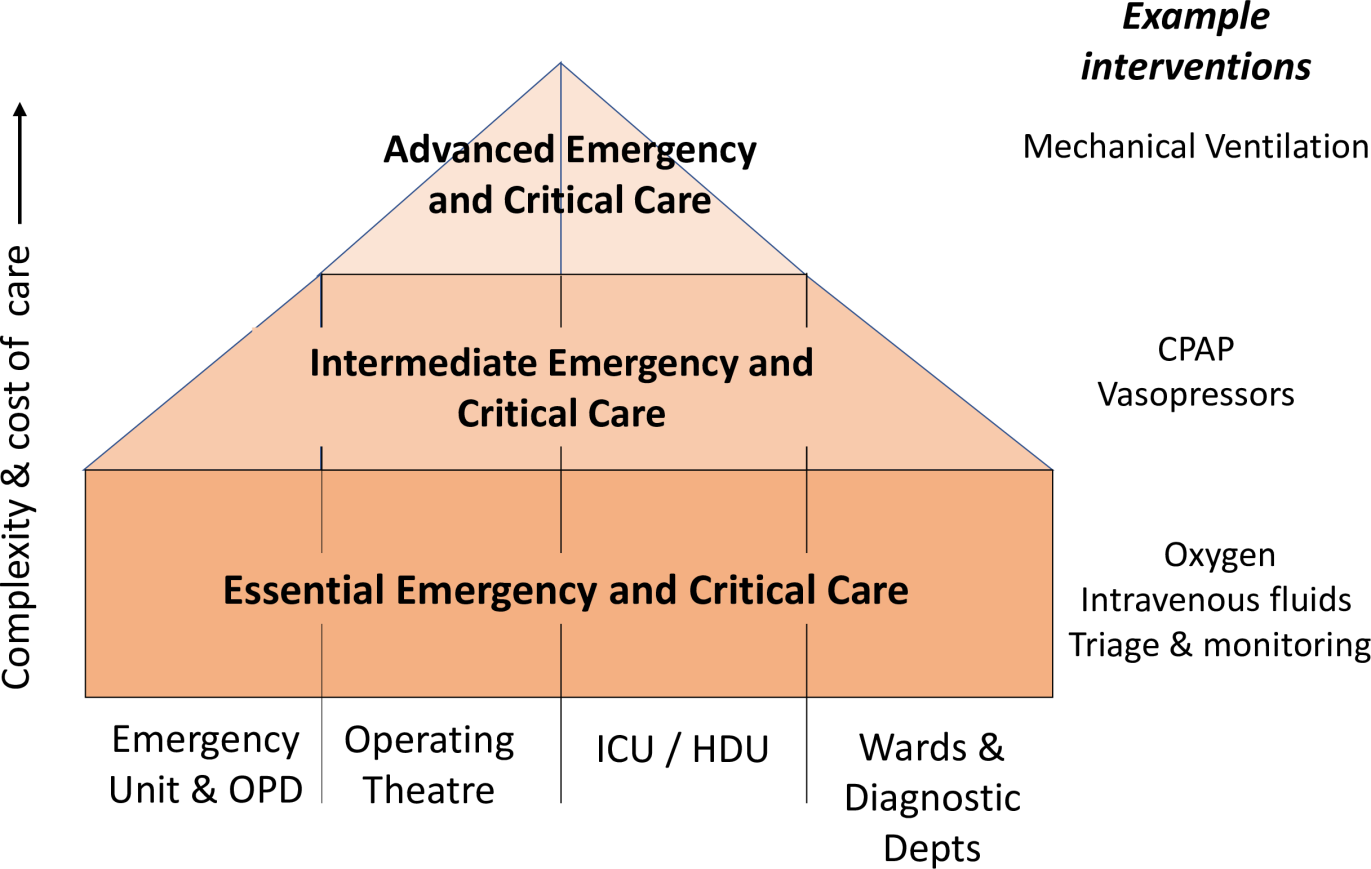
Conceptual model outlining how we might structure care for those with critical illness. At the base of the pyramid, all facilities or departments (including outpatient (OPD) and diagnostic departments (depts)) should implement processes to identify critical illness, monitor patients and deliver essential interventions such as oxygen and intravenous fluids. Delivery of intermediate or more advanced care needs more specialised settings, for example, use of continuous positive airway pressure (CPAP) or even mechanical ventilation. These might be provided in distinct parts of a larger facility or require referral to another facility. The effectiveness of the whole system will depend on the quality of care at the foundational level, sustaining quality care during any transitions across system levels and locations and quality of care at higher system levels. ICU, intensive care unit; HDU, High Dependency Unit.

Given this backdrop, the team was specifically interested to learn lessons of relevance to all hospitals to guide those demanding improvements in care for emergencies and the seriously ill with a particular focus on ‘Essential Emergency and Critical Care’ (EECC).^7 8^ The basic principles of EECC are that a relatively small set of apparently simple interventions delivered in a timely fashion to patients with critical illness could save many lives irrespective of where patients are being cared for in a facility. Thus, not only emergency and intensive care units (ICUs) but also outpatient areas, wards, operating theatres and even areas providing diagnostic services all require EECC capacity and capability. EECC therefore forms the base of health facilities’ pyramid of responses to care for critical illness and has been defined in full elsewhere (figure 1).^7^ It requires that all facilities or hospital departments should have basic resources and appropriately skilled personnel, while fewer settings in a single facility or at a separate facility based on planned referral strategies might offer intermediate-level care (eg, use of continuous positive airway pressure) or even more advanced care (eg, use of mechanical ventilation). Severe COVID-19 was both a specific challenge to the provision of hospital care and an exemplar of critical illness in general. Here, we outline the lessons learnt by our multidisciplinary team as the COVID-19 pandemic unfolded. Much needed strategies to improve care for emergencies and critical illness should be guided by such evidence to avoid mistakes being repeated.^8^

## Lessons learned

### How ready were hospitals to deliver EECC during the COVID-19 crisis?

Our studies in Kenya and Tanzania conducted in late 2020 and in 2021 found that hospitals were generally poorly prepared to cater to the basic needs of those with critical illness.^9 10^ Evaluations of equipment and the human and material resources for dealing with acute, severe illness highlighted considerable variability between hospitals in such inputs and were generally lacking in Tanzania. Even in better-resourced settings, there were major deficiencies in local organisational arrangements and clinical processes.^10^ For example, national referral hospitals described uniform triage processes within their emergency departments, but triaging systems in all hospitals’ wards and in the emergency departments of general hospitals were largely absent.^9 10^ Importantly, in both countries, even if hospitals had equipment and materials required to institute EECC, they typically lacked the management systems and routines for the proper deployment and use of these resources. For example, piped oxygen systems installed in some locations were often ‘present’ but not fully functional and staff abandoned them, reverting to older habits of relying on bottled oxygen. Worryingly, even in the midst of the COVID-19 pandemic, infection prevention and control measures remained weak. For example, in Kenya only two out of five facilities had protective face masks and aprons widely available, compounding wider problems of overcrowded, poorly maintained hospital buildings often with limited water provision.^9 10^ Our findings in Kenya and Tanzania are consistent with those from other low-resource settings.^11^

EECC requires that all staff are able to identify critical illness and provide the most basic immediate care that a critically ill patient would need. In Tanzania, personnel with any relevant training were available in the facilities on average less than 50% of the time.^10^ Skills deficits exacerbated substantial human resource shortages across both countries, particularly in more rural or smaller hospitals; they are a fundamental limitation to the proper treatment of the critically ill patients. Examples included 1 clinical officer (a form of non-physician clinician) attending to approximately 16 patients at one time, 1 nurse looking after 22 patients including some with critical illness and 1 doctor attending to 51 patients.^9^ Interestingly, such extreme staff to patient ratios could not be attributed to surges in COVID-19 admissions, as data actually suggest this did not occur.^12 13^ Not surprisingly, staff described frequently feeling‘overwhelmed’. The lack of skilled staff and high patient-to-clinician ratios emphasise the need to prioritise improving staffing as well as organisational processes, if facilities are to deliver effective EECC.^11 14^

### Moving beyond resources: providing continued effective care

Effective management of critical illness including COVID-19 requires quality care sustained over hours or days. This includes periods spent in emergency departments, during transfers, during waits in diagnostic departments for investigations and sometimes prolonged periods spent in wards. The quality of continuous care, often reliant on nurses, has been much less well examined in low-resource settings, although some prior work suggests inadequacies.^15^ Our team used an approach based on following specific patient journeys in Kenya and Tanzania to examine this. This simple strategy highlighted how errors and dangerous delays were rooted in a lack of planning for providing even the basic forms of care encompassed in EECC uniformly across hospital settings.^16^ Inadequate organisational responses compounded the material and human resource shortages outlined above. For example, resulting in a critically ill patient with dangerously low oxygen saturation deteriorating due to delays in receiving timely care.^16^ Similarly, in departments such as pharmacy, laboratory and radiology, all suffering from personnel and material shortages, the delays in receiving diagnostic or therapeutic interventions compromised EECC. For example, resulting in extensive periods when critically ill patients were unobserved in reception areas. Patient journeys particularly highlighted the atomised nature of care in the hospitals we studied. In some cases, departments seemed to have an almost adversarial relationship, wanting to push out as many patients as possible while restricting new arrivals. These perpetual stresses combine to create a general sense of resignation and organisational norms that are likely to be highly resistant to positive change (a situation we describe as ‘arrested development’).^9^

Combining insights from patient journeys, formal interviews and direct observations allowed us to develop a broader conceptual understanding of the process challenges to delivery of quality EECC in Kenyan and Tanzanian hospitals. We propose it is helpful to think of three distinct areas of concern in any hospital location; how it receives, sustains and transfers any patient with critical illness. The receipt of patients into a facility or department was rarely well structured. For example, systems to identify that a patient waiting in a queue has probable sepsis and low blood pressure or other features of incipient or manifest critical illness were absent. Frequently, establishing the patient’s ability to pay was prioritised above such triage. Maintaining vigilance (monitoring and reassessment) even by assessing basic vital signs or monitoring ongoing treatment (eg, fluid or oxygen therapy) was often lacking. Worryingly, this was apparent during extended stays in wards as well as during shorter periods in emergency or other hospital departments. Although this often reflects staff shortages, functional systems for prioritising patients so that time is devoted to reassessment and care of the most ill were rare. Lastly, the flow of patients within and between departments was often problematic and reliant on family members if present. It could lead to long periods within a facility where critically ill patients could deteriorate while waiting to move to the next stage of care.^16^ In fact, issues as fundamental as a lack of any unified understanding of what comprises ‘critical illness’ among health workers also contribute to communication failures and suboptimal organisational processes.^17^ In sum, the absence of structured organisational systems means in many hospitals, the provision of EECC becomes contingent on the awareness, capacity and motivation of individuals to do what they consider best in very difficult circumstances.

### Efforts to improve care for the critically ill patients: the global and national response

Concerned by the catastrophic potential of COVID-19, countries had to act. With rich countries’ ICUs flooded with patients early in the pandemic, initial global attention focused on how to rapidly scale up advanced respiratory support in low-resource settings.^18^ The result was national governments, multilateral agencies and private philanthropy urgently sourcing and distributing equipment such as ventilators.^19 20^ Unfortunately, in our opinion, this response ignored prior information from existing health facility readiness assessments and research.^3 6 21^ While it was true that Kenya and Tanzania had very few ventilators, it was also known they had an extreme shortage of medical, nursing and other specially trained personnel able to manage ventilated patients.^20 22^ Seconding personnel from another hospital area to address gaps in the critical care workforce and attempting to upskill them with extensive virtual training risked more pronounced workforce deficits elsewhere. This actually threatens provision of EECC more widely, especially as emergency hiring programmes to increase staffing levels were typically short-lived.^20^

Recognition that attention had to be paid to apparently basic forms of care for critically ill patients with COVID-19 rather than advanced respiratory support was slow. In our facility surveys, sometimes many months after the onset of the pandemic, access to oxygen remained low.^9 10^ Indeed, it took many months for the focus of global support to shift to improving access to medical oxygen, a critically important agenda known to have been neglected for many years.^23^ Even now though, the focus is on solving the ‘hardware’ or equipment issues and training. The logical simplicity that one can solve the problem of acute care by providing oxygen and other basic materials belies the challenges faced by health systems. Attempts to strengthen overall staffing levels and organisational processes remain neglected. This challenge is compounded by the interpretation of health facility assessment or readiness surveys (including the Service Availability and Readiness Assessment tools). These identify hospitals that completely lack essential inputs, but they do not provide any real sense of whether a hospital and its staff have the minimum functional level of inputs combined with needed individual and organisational capabilities to provide safe, effective care.^9 10 16 24^ Training specialist ICU staff will not paper over the much wider cracks in the availability of well-trained general nurses and clinicians that undermines delivery of quality care to the critically ill patients and all others in need. Moreover, specialist professionals often champion the needs in their specific field of expertise, leaving few to champion prioritisation of more basic forms of care such as EECC as a foundational approach.^7 25^

Our team also examined the potential cost-effectiveness of improving care for critical illness in low-resource settings and specifically improving coverage of EECC. These studies suggest that EECC can be both low cost-effective and highly cost-effective. In Tanzania and Kenya, the cost of current advanced critical care conducted in ICUs with specialised staff, facilities and technologies is nearly ten times than that of EECC.^26^ In Tanzania, preliminary analysis has found that the probability of EECC provision being cost-effective is greater than 95% while more advanced critical care is not cost-effective based on commonly used thresholds.^26–29^ While these estimates provide a good benchmark, they have limitations; observational studies on the clinical effectiveness of EECC are not yet available. However, a focus on delivering quality EECC at scale would also promote equity, making it a priority for inclusion in universal health coverage strategies.^30^

**Figure 2:**
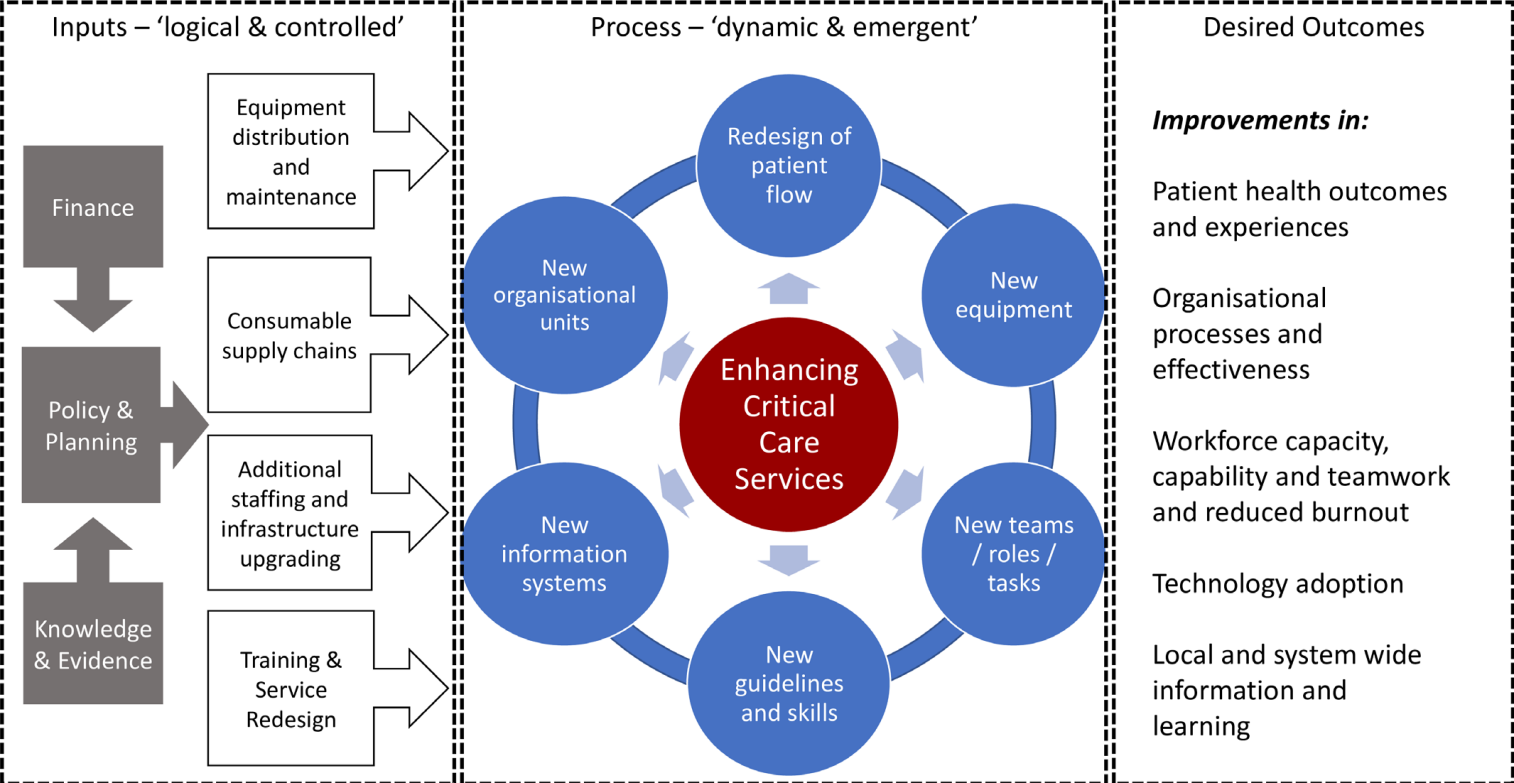
Changing complex health systems at scale is hugely challenging. For simplicity, we present the broad efforts required to introduce EECC and more advanced levels of critical care in health facilities employing the familiar input, process, output or outcome framing. Key inputs are often seen as complicated but quite logical; however, these need to well beyond provision of technologies and should be supported by careful planning and financing as improvement efforts may well require rethinking of service provision and sustained supply of resources and equipment maintenance. The process of change is often reduced to a focus on new guidelines and skills training around new technologies but is much more complex. Process changes may involve adaptation to each specific context and may result in new ways to organise services (eg, allocation of specific spaces to the critically ill patients), new health worker roles and new information needs or resources (eg, new patient charts or handover tools). Attention must be paid to these dynamic and emergent processes that will require local leaders to take on change management roles if systems are to achieve better patient, staff and system outcomes including the improved teamwork that facilitates adoption and effective use of any new technologies.

Our findings highlight the foundational quality challenge of delivering the right care, at the right time to the right individual across all parts of hospitals at scale.^1–5^ This is not simply a problem of providing the right equipment or materials nor of providing the right life-support training to build workforce competence, although these are needed. Saving lives threatened by critical illness requires us to tackle wider system challenges.^31^ These include prioritising the care that can impact most patients and lead to the best outcomes with the fewest resources. Improving general patient to staff ratios is needed as we create leaders with the knowledge, technical and importantly service management skills to oversee the structural and cultural organisational changes that are needed to sustain quality care throughout hospital journeys. This vital service improvement capability is often neglected in low-resource health systems. However, the response to severe COVID-19 was a reminder of the carefully planned and continuous work required to redesign and improve services (figure 2). The COVID-19 pandemic revealed major gaps in policy and planning in all countries and inadequacies in supply chains, staffing, technologies and financing to support these in most. The global response aimed to tackle some of these, often temporarily in the case of human resources, but paid much less attention to developing the skilled service improvement and implementation work that must span multiple facility locations, teams and processes that are essential to achieve the changes our findings indicate are needed (figure 2). To be effective, these much needed system changes requires people who can negotiate and manage complex-dependent and context-dependent challenges. These include the high levels of psychological distress and burnout in the workforce that threaten quality care, particularly in low-resource settings.^32^ While leadership from emergency and critical care specialists is likely to be important, does currently offered specialist training in critical care build these much needed skills?

In summary, our multidisciplinary team found that hospitals in Kenya and Tanzania, as in other low-resource settings, may have some of the essential resources they need to offer EECC. Although each study has limitations, and even a set of studies cannot claim to be a full and accurate representation of complex systems, taken together our work indicates that we need to go beyond considering EECC as a package of life-saving interventions and think of it as a continuous process. Considered this way, EECC is almost a state of mind requiring a form of systemic vigilance and response, while ensuring the care that is most needed is prioritised first. High-income countries have invested over decades in personnel and their skills, improvements in tools and organisational processes. In some places, this even includes dedicated ‘floor level’ management to promote timely action. These system responses are almost completely absent in Kenya and Tanzania. They must be carefully developed alongside efforts to tackle basic infrastructure and resource gaps. Addressing only the problem of technical inputs by offering training and technologies without addressing system challenges seems little more than building sandcastles on a beach and ignoring the reality of the incoming tide.

## Conclusion

Making wise investments should be a priority of countries and the global community. Reactive responses in the first months of the pandemic resulting in a focus to supply advanced equipment for critical illness were often inappropriate.^19 20 28^ We propose a five-part strategy to guide development and implementation of EECC as part of the broader strengthening of health systems to address the needs of the critically ill patients. This strategic approach draws on well-established organisational change frameworks and more recent global health thinking and is outlined in table 1.^33–36^ The strategy recognises the inherent complexity of health systems and therefore that achieving meaningful change goals requires alignment of efforts combined with sustained, skilled day-to-day leadership and management across levels of the health system. The five parts are therefore not a proscriptive recipe for action but a set of guiding principles that could inform more context-specific planning and action. They include first a need to recognise that: (1) delivering EECC is a system not a clinical problem, (2) countries need first to diagnose the extent of their problems, (3) a driving coalition and consensus, spanning all levels of the health system, will be required to achieve change, (4) new resources, energetic leadership and carefully considered incentives (typically non-financial) will be required to support change and (5) if the driving coalition is to institutionalise EECC as a key feature of quality healthcare key actors will need to be empowered so they can sustain change efforts over periods longer than is typical of many programmatic initiatives. Such a strategy requires better pre-emptive generation and use of data and local knowledge to guide context-specific planning and responses. This itself requires a greater focus on national capacity to direct, coordinate, mobilise and monitor investments that optimise longer-term system strengthening and promote equity. Actors from across the health system also need to prioritise interventions and be clear about the basic standard of care that is the initial focus. This may mean that specialists from across acute care disciplines need to develop the skills to understand and manage care for the critically ill patients, as a complex process spanning whole facilities.^37 38^ This includes lower level facilities, as care for the critically ill patients is not just the preserve of critical care specialists in their critical care units. Researchers can support these efforts by helping to embed continuous learning in such endeavours and maintaining a long-term focus on institutionalising high-quality care for all, everywhere.

**Table 1:** A five-part strategy drawing on organisational change frameworks to guide design and implementation of national efforts to deliver EECC at scale that acknowledges the long-term, sustained and multistakeholder efforts that will be needed^33^

1	**Recognise care for the critically ill patients and provision of EECC at scale as a system problem,** not a discipline-specific clinical problem mainly characterised by technology deficits.
2	**Diagnose and prioritise the full extent of the health system challenges** preventing delivery of high-quality EECC to all by bringing together data and providers from all system levels.
3	**Develop a driving coalition and consensus, spanning all levels of the health system**, around a feasible strategy for improving care for the critically ill patients beginning with setting standards for EECC in facilities as part of universal health coverage before progressing to expansion of high dependency and subsequently specialist emergency and intensive care. This should be linked to a clear and compelling vision that is widely communicated to all and supported by important stakeholders including specialist professionals with clear roles for any centres/personnel with existing expertise to support implementation of EECC at scale.
4	**Mobilise new resources, inject energy and incentivise the process of change** through measures such as: Developing a set of personnel, potentially in the form of a network, with the mandate, skills and resources to work with site-specific teams to identify the challenges facilities face in provision of EECC and formulate context-specific facility improvement plans. Providing additional funding to implement the improvement plans including the capacity to address critical staff shortages, reorganise patient pathways, introduce local facilitators to do the day-to-day work of change and upgrade skills and basic equipment (reallocation of existing funding from one area to this new area is unlikely to be sufficient nor effective and may cause harm to another part of the system). Ongoing monitoring and supportive supervision to help drive local change processes, enable cross-site learning, highlight successes and sustain advocacy for the multiyear support and improvements needed at system level.
5	**Empower the driving coalition to institutionalise EECC as a key feature of quality healthcare** recognised by all health professionals over a period of 5–10 years including: Ensuring that basic skills are effectively developed as part of preservice health worker education and reinforced through systems of continuous professional development. Ensuring that local health-care managers and specialist health workers themselves have the skills to diagnose local system problems and implement improvements so that leadership and responsibility for providing high-quality EECC are distributed while continuously supported by higher level expertise. Reviewing progress to adapt and improve the implementation strategy and continue to mobilise new resources while ensuring that high quality EECC for all remains the goal.

EECC, Essential Emergency and Critical Care.

## Data Availability

There are no data in this work.
